# Supplementary material for: Lysosome-dependent nutrient scavenging underlies stress adaptation during epithelial-to-mesenchymal transition
Source: bioRxiv. 2025 Sep 23:2025.09.22.677807. Preprint. [Version 1] doi: 10.1101/2025.09.22.677807 (PMC12485954; doi:10.1101/2025.09.22.677807)
Supplement: Supplement 1 [file NIHPP2025.09.22.677807v1-supplement-1.pdf]

bioRxiv preprint doi: https://doi.org/10.1101/2025.09.22.677807; this version posted September 23, 2025. The copyright holder for this preprint (which was not certified by peer review) is the author/funder, who has granted bioRxiv a license to display the preprint in perpetuity. It is made available under aCC-BY 4.0 International license.

Extended Data Figure Legends

Extended Data Fig. 1 Mutant ERK2 signaling is associated with EMT signatures found in metastasis models. a, Immunoblot of MCF10A vector control cells and those expressing ERK2- WT, Y261A, and D319N collected over 9 days of doxycycline stimulation (0.25 ng/ml). Corresponding to images in **Fig. 1b**. **b**, Mutation diagram of ERK2/MAPK1 common docking site mutation occurrence of non-redundant pan-cancer data available on cBioPortal (101,480 patients). **c**, Three-dimensional rendering of the common docking site of ERK2. Indicated residues correspond to Human numbering along with Rat (constructs used in this study) and are indicated by arrows and colored magenta. **d**, Immunoblot of MCF10A vector control cells and those expressing ERK2 common docking (CD) site mutants found in tumors. **e**, Temporal heatmap of gene expression in MCF10A $ERK2^{D319N}$ expressing cells compared to controls. The gene set was previously identified by Padua and collogues (ref. 24), as the TGF- β response associated with breast tumor lung metastasis. Data represent log2 fold-changes. **f**, Enrichment analysis of transcripts that increase in expression and overlap with Jin and collogues (ref. 25). **g**, Enrichment analysis of transcripts that decrease in expression and overlap with Jin and collogues (ref. 25). **h**, Temporal heatmap of EMT transcription factor gene expression in MCF10A $ERK2^{D319N}$ expressing cells compared to controls.

Extended Data Fig. 2 Convergence of ERK2 and TGF-β signaling in EMT. a, Immunoblot of MCF10A TGF-β1 (5 ng/ml) stimulated cells (Day 4), treated with 1 μM MEK inhibitor Selumetinib (MEKi) for 24h. **b**, Immunoblot of MCF10A with knockdown of SMAD2, SMAD3, or SMAD4, and effect on PAI-1 expression in MCF10A $ERK2^{D319N}$ expressing cells (Day 3). **c**, Matrigel invasion assay corresponding to samples in **b** (n = 3). **d**, Immunoblot of SMAD2, SMAD3, and corresponding phospho-deficient mutants in $ERK2^{D319N}$ expressing MCF10A cells. **e**, Immunoblot of SMAD2, SMAD3, and corresponding phospho-deficient mutants in TGF-β1 (5 ng/ml) stimulated MCF10A cells. **f**, Brightfield images corresponding to samples in **d**. **g**, Brightfield images corresponding to samples in **e**.

Extended Data Fig. 3 Lysosome induction in EMT. a, Venn diagram and **b**, heatmap of differentially expressed proteins in $ERK2^{D319N}$ expressing and TGF-β1 stimulated cells (5 ng/ml). **c**, Enrichment analysis of statistically significant (p>0.001) up and down-regulated proteins in the two models of EMT. **d**, Fluorescence microscopy of DQ-BSA in $ERK2^{D319N}$ expressing cells treated with 100 nM concanamycin A

bioRxiv preprint doi: https://doi.org/10.1101/2025.09.22.677807; this version posted September 23, 2025. The copyright holder for this preprint (which was not certified by peer review) is the author/funder, who has granted bioRxiv a license to display the preprint in perpetuity. It is made available under aCC-BY 4.0 International license.

(CMA) or 50 μM chloroquine (CQ). **e-f**, Immunoblot and corresponding DQ-BSA fluorescence integrated density in MCF10A cells expressing ERK2^{WT} or ERK2^{D319N} stimulated with TGF-β1 (5ng/ml) (n = 3). **g**, Enrichment clustergram of significantly increased proteins from proteomics of ERK2^{D319N} expressing cells on day 3. Red bars (top) correspond to the combined score of listed KEGG terms.

Extended Data Fig. 4 Macropinocytosis in EMT. a, Live cell fluorescence microscopy of Alexa Fluor 647 conjugated BSA uptake in MCF10A cells expressing EGFP-2xFYVE.

Extended Data Fig. 5 Effects of mTOR-inhibition on invasion and lysosome inhibition on EMT cell toxicity. a, Matrigel invasion assay of MCF10A cells expressing ERK2^{D319N} or stimulated with TGF-β1 (5 ng/ml) and treated with 250 nM Torin at the time of seeding in the Boyden chamber. Cell invasion was analyzed after 24 h (n = 3). **b**, Annexin V staining of MCF10A control cells and those undergoing EMT starved of essential AA and glutamine (-EAAQ) for 48 h in the presence of 100 nM concanamycin A (CMA) (n = 3).

Extended Data Fig. 6 c-MYC opposes EMT associated amino acid transport changes and metabolic switch. a, Immunofluorescence of LAMP2 in MCF10A undergoing EMT with c-MYC expression. Data collected over 4-5 fields, n ≥ 23 cells per group. **b**, Immunofluorescence of CD63 and phalloidin in MCF10A undergoing EMT with c-MYC expression. Data collected over 2 fields, n ≥ 36 cells per group. **c**, Immunofluorescence of LAT1/SCL7A5 in MCF10A undergoing EMT with c-MYC expression. Data collected over 3 fields, n ≥ 100 cells per group. **d**, Glycolysis stress test profile indicating changes to the extracellular acidification rate (ECAR) of cells expressing control, ERK2^{D319N}, c-MYC or ERK2^{D319N} with c-MYC (Day 3). **e**, Mitochondrial stress test profile indicating changes to the oxygen consumption rate (OCR) of cells expressing control, ERK2^{D319N}, c-MYC or ERK2^{D319N} with c-MYC (Day 3).

bioRxiv preprint doi: https://doi.org/10.1101/2025.09.22.677807; this version posted September 23, 2025. The copyright holder for this preprint (which was not certified by peer review) is the author/funder, who has granted bioRxiv a license to display the preprint in perpetuity. It is made available under aCC-BY 4.0 International license.

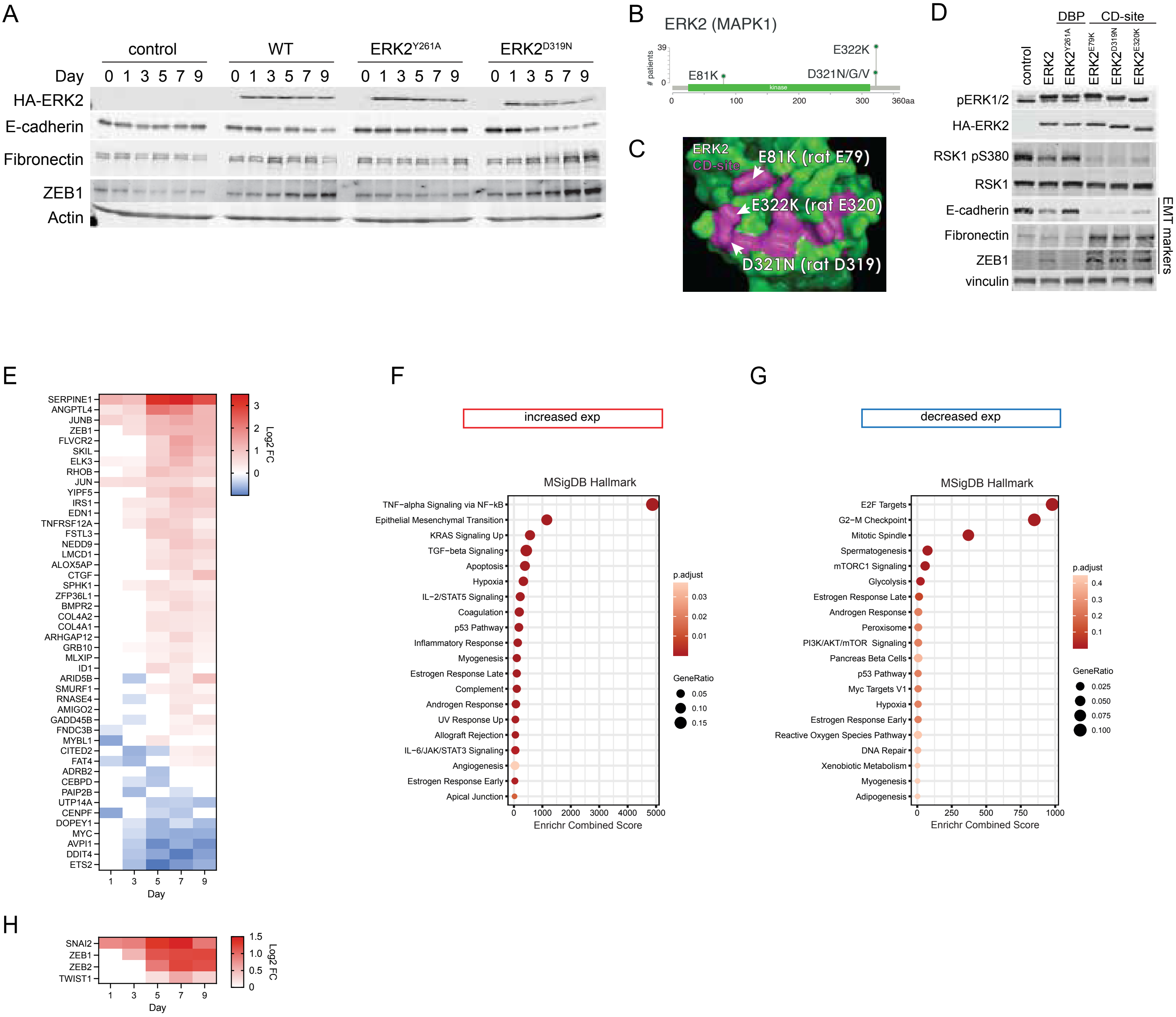

Supplementary Figure 1

bioRxiv preprint doi: https://doi.org/10.1101/2025.09.22.677807; this version posted September 23, 2025. The copyright holder for this preprint (which was not certified by peer review) is the author/funder, who has granted bioRxiv a license to display the preprint in perpetuity. It is made available under aCC-BY 4.0 International license.

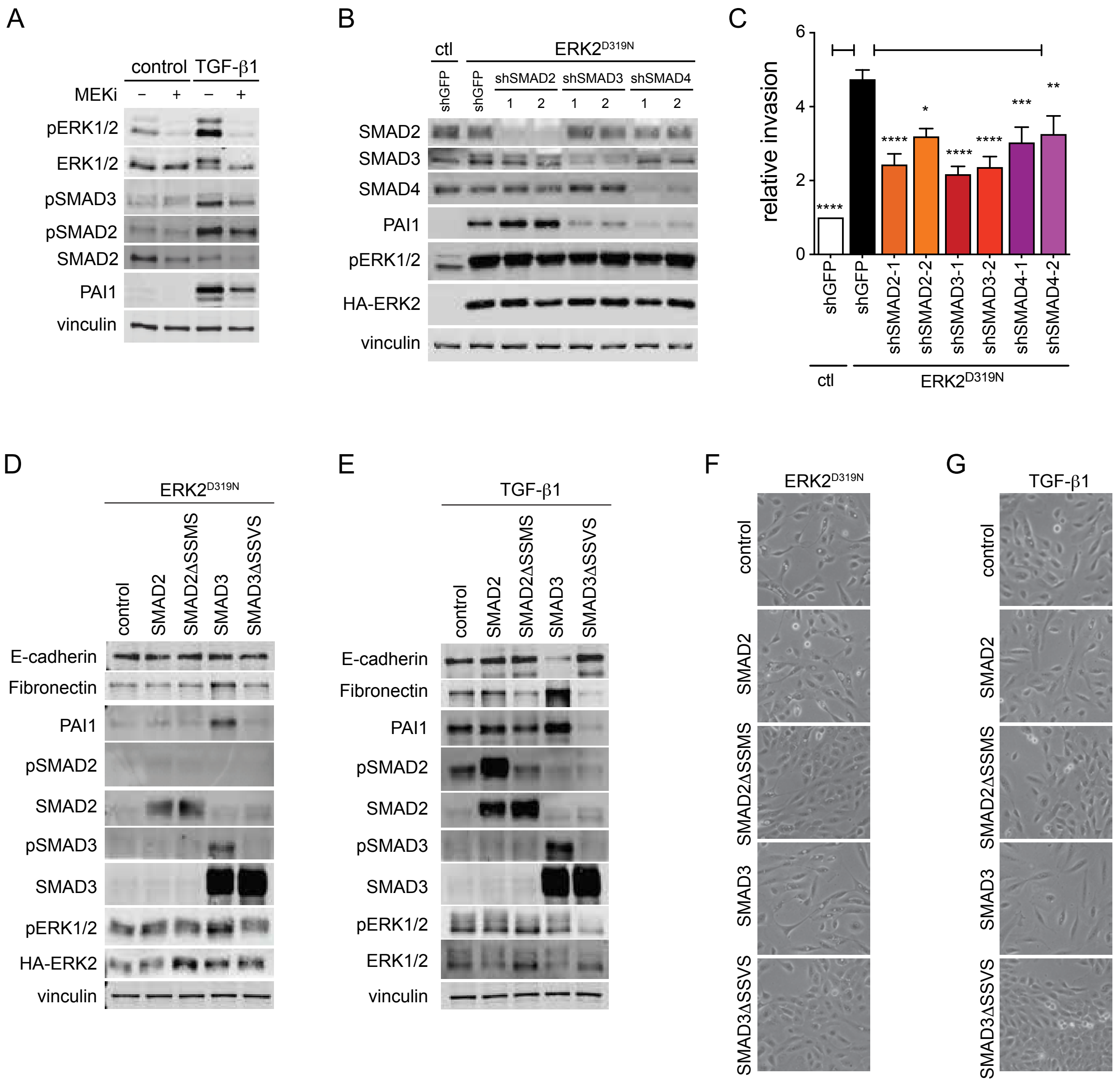

Supplementary Figure 2

bioRxiv preprint doi: https://doi.org/10.1101/2025.09.22.677807; this version posted September 23, 2025. The copyright holder for this preprint (which was not certified by peer review) is the author/funder, who has granted bioRxiv a license to display the preprint in perpetuity. It is made available under aCC-BY 4.0 International license.

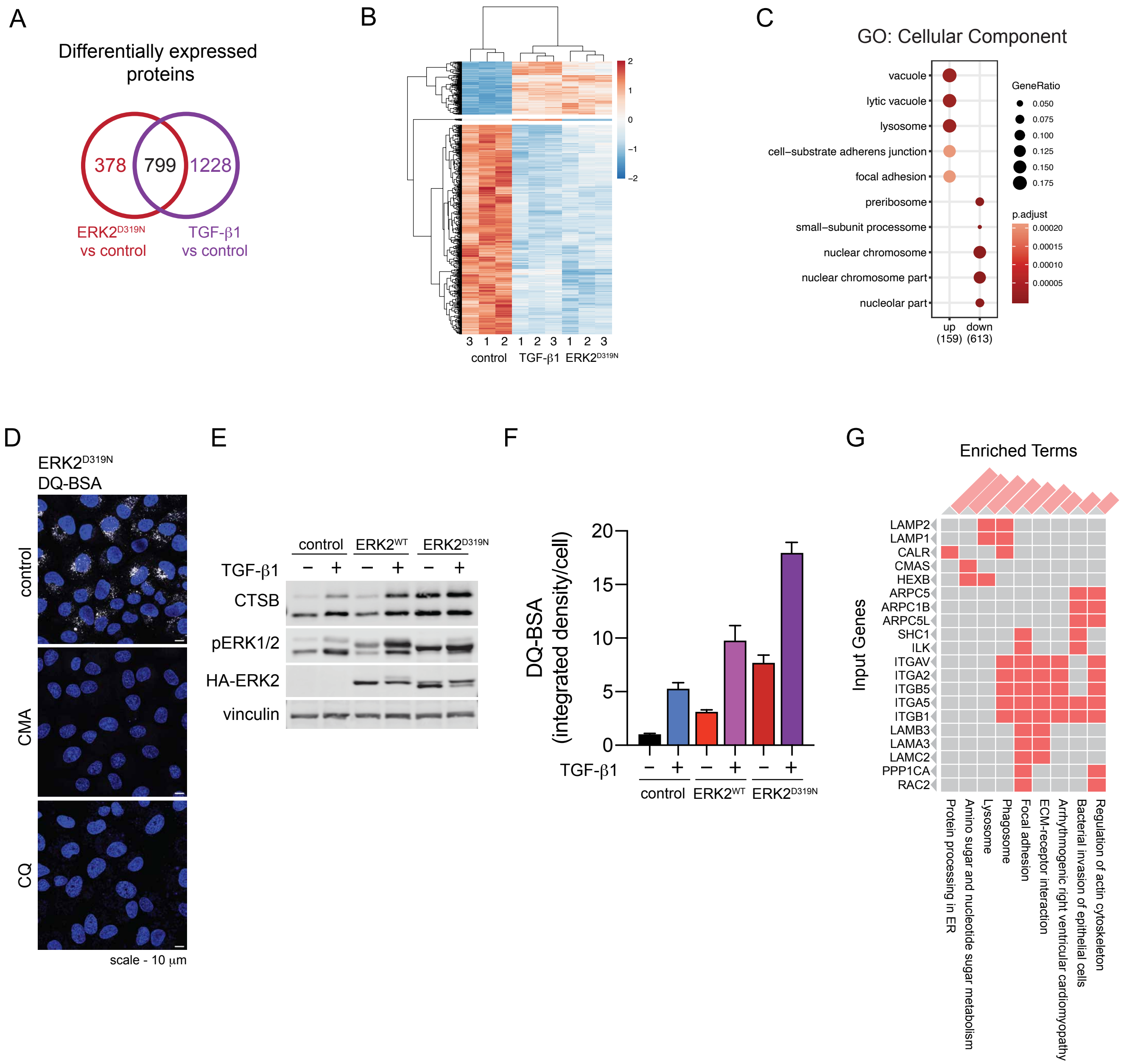

Supplementary Figure 3

bioRxiv preprint doi: https://doi.org/10.1101/2025.09.22.677807; this version posted September 23, 2025. The copyright holder for this preprint (which was not certified by peer review) is the author/funder, who has granted bioRxiv a license to display the preprint in perpetuity. It is made available under aCC-BY 4.0 International license.

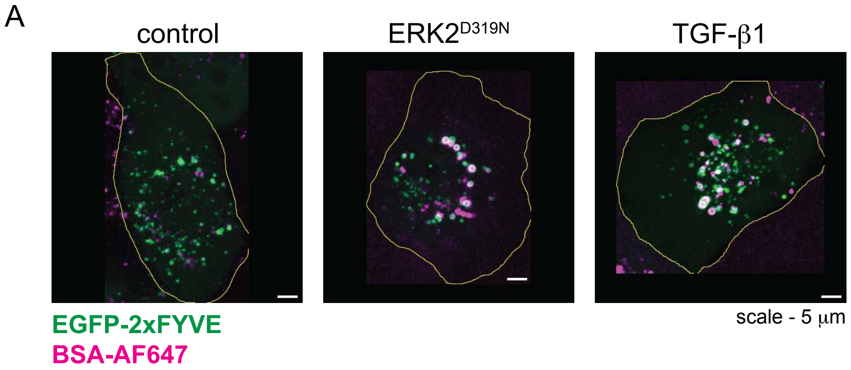

Supplementary Figure 4

bioRxiv preprint doi: https://doi.org/10.1101/2025.09.22.677807; this version posted September 23, 2025. The copyright holder for this preprint (which was not certified by peer review) is the author/funder, who has granted bioRxiv a license to display the preprint in perpetuity. It is made available under aCC-BY 4.0 International license.

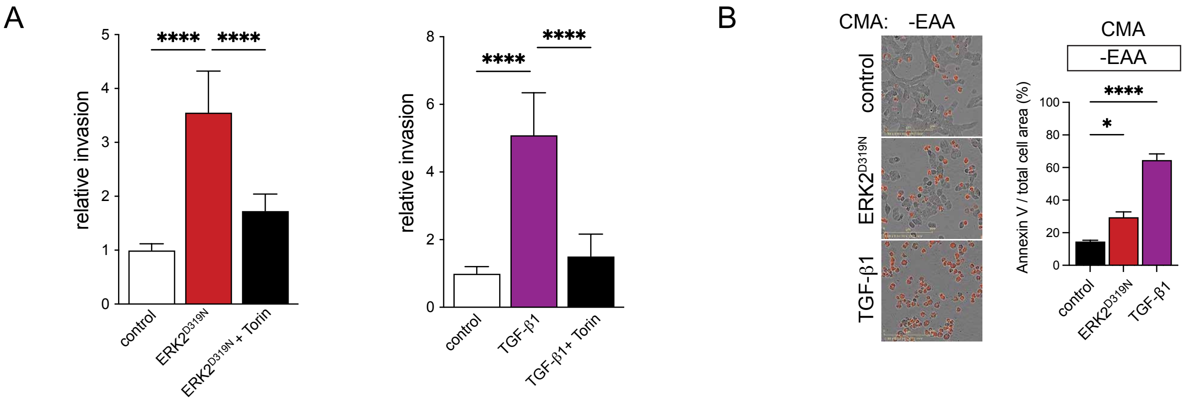

Supplementary Figure 5

bioRxiv preprint doi: https://doi.org/10.1101/2025.09.22.677807; this version posted September 23, 2025. The copyright holder for this preprint (which was not certified by peer review) is the author/funder, who has granted bioRxiv a license to display the preprint in perpetuity. It is made available under aCC-BY 4.0 International license.

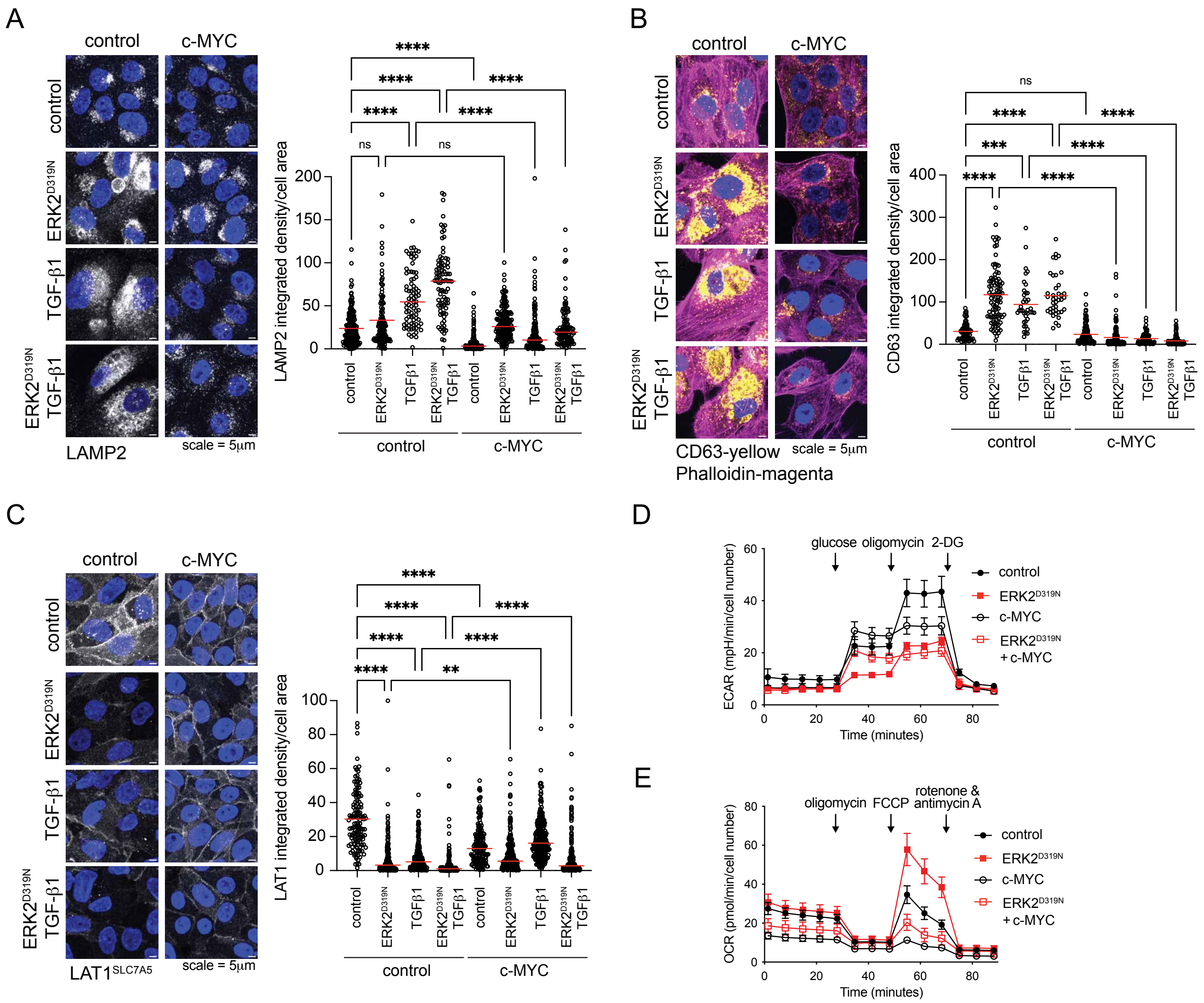

Supplementary Figure 6
